# Modified percutaneous needle aponeurotomy for Dupuytren’s disease: case series with functional outcome

**DOI:** 10.1007/s12306-025-00899-5

**Published:** 2025-03-31

**Authors:** Anil K. Bhat, P. K. Navaneeth, G. Mithun Pai

**Affiliations:** 1 Department of Hand Surgery, Kasturba Medical College, Manipal, India; 2 Manipal Academy of Higher Education, Manipal, 576104 India

**Keywords:** Percutaneous, Needle, Fasciotomy, Recurrence, Contracture, Dupuytren

## Abstract

**Purpose:**

Over the past three decades, percutaneous needle aponeurotomy (PNA) for Dupuytren’s disease has become increasingly prevalent and offers numerous potential benefits. However, significant rates of recurrences are observed in literature. We aimed to evaluate the effectiveness of our technical modifications of percutaneous needle aponeurotomy that significantly separates the cord in the palm and digits, thereby minimizing recurrences.

**Methods:**

We treated 23 consecutive patients with our modified technique. We use a larger-bore needle in the dorsovolar direction which involves a more controlled extensive disruption of the cord under local anesthesia. The mean total passive extension deficit (TPED) and the Dupuytren’s contracture-specific Unité rhumatologique desaffections de la main (URAM) scores were calculated at final follow-up.

**Results:**

Twenty-three patients underwent the procedure, involving 28 affected hands and 38 fingers, including 22 ring fingers, 6 little fingers, and 10 middle fingers. The average follow-up period was 22 months, ranging from 12 to 28 months. At presentation, the mean total passive extension deficit TPED was 50°. At the time of the final follow-up, the mean (TPED) was 10°, with a mean percent correction of 83% which was found to be statistically significant. This included 82% correction at the metacarpophalangeal joint and 81% at the proximal interphalangeal joint. Only four experienced recurrences, accounting for 14.2% of the total with a mean TPED of 30°. Lower URAM score indicated a significant short-term functional improvement.

**Conclusion:**

Our modified PNA technique enhances cord division in the palm and fingers, leading to improved contracture correction and lower recurrence rates. However, further studies with larger cohorts and control groups are needed to validate these findings.

## Introduction

**Fig. 1:**
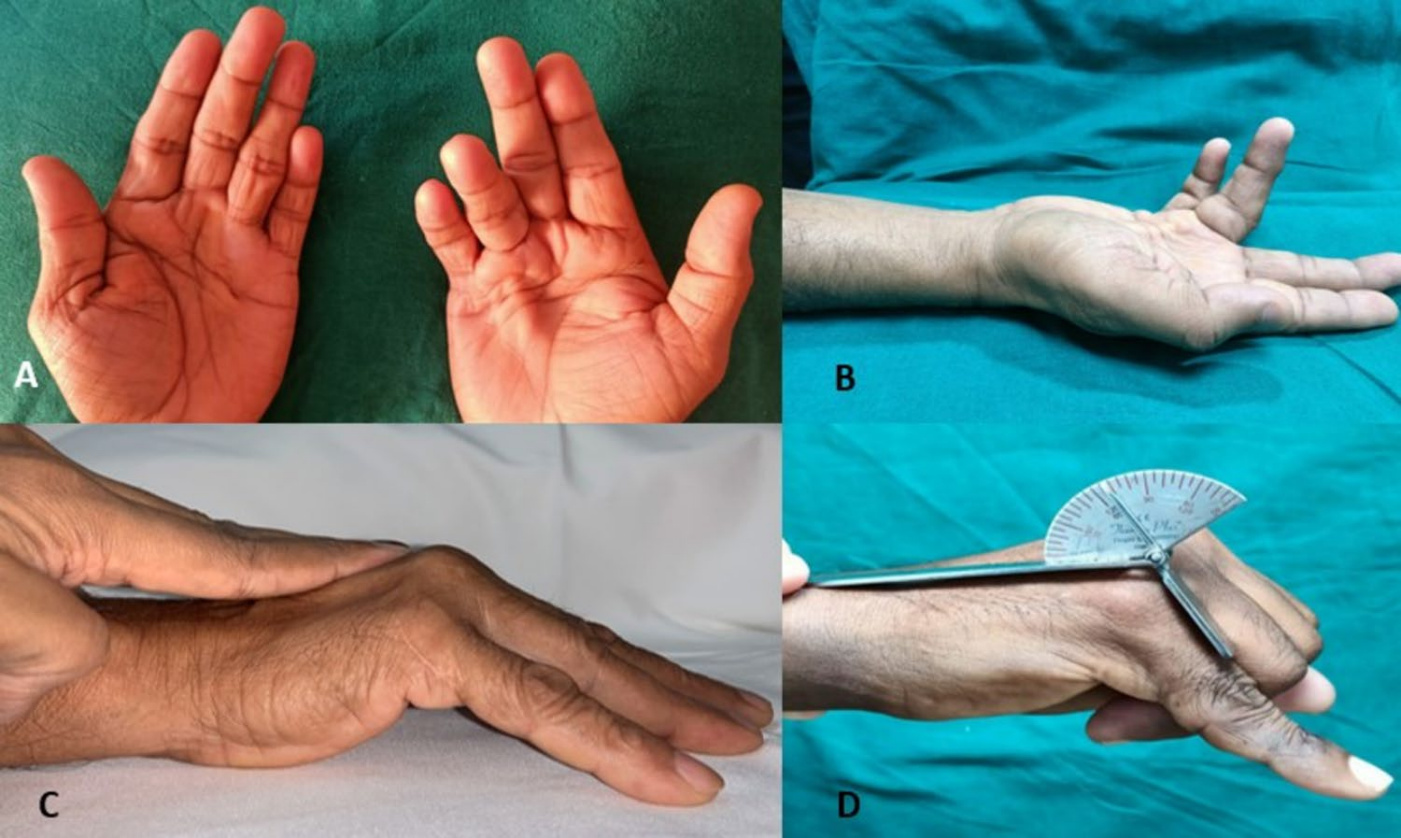
**A** and **B** Dupuytren’s contracture involving predominantly the ring finger and partly the little finger, C) calculating total passive extension deficit using finger goniometer, and D) preoperative positive table top test

Dupuytren’s disease, a benign fibroproliferative disease of the palmar fascia, can cause significant finger flexion contractures and limit hand function. (Fig. 1 A, B) Limited fasciectomy (LF) is the most commonly employed treatment for Dupuytren’s contracture and is considered the most effective method [1]. Percutaneous needle aponeurotomy (PNA) has gained increasing popularity as a daycare procedure due to its minimally invasive nature and ability to be performed under local anesthesia [1, 2]. Using intradermal anesthesia, monitoring the distal sensibility throughout the procedure is possible, preventing damage to the digital nerves [2]. Monitoring needle movement with active finger flexion provides a rough estimate of the proximity of the needle to the flexor tendon, reducing the risk of tendon injury.^2^ Skin tears, reported in as many as 68% of cases, appear to be the primary yet minor concern that is treatable [1]. Studies suggest that needle aponeurotomies have higher recurrence rates than open fasciectomy [1]. Recurrence following needle aponeurotomy may have distinct consequences than that following open surgery. In a diligently conducted randomized controlled trial, Van Rijssen reported that 45 patients (84.9%) experienced recurrence after percutaneous needle aponeurotomy performed by a classical pendulum motion technique using a 25 G hypodermic needle. However, 26 out of 45 patients with recurrence chose to repeat the procedure, unlike those who underwent open fasciectomy. [3] We have implemented technical modifications in our technique of needle aponeurotomy that allow significant widespread separation of the cord in the palm and fingers to improve contracture correction, particularly at the PIP joint, with fewer complications and recurrences. This study reports the outcomes of a pilot cohort comprising 23 patients who underwent a modified percutaneous needle aponeurotomy (PNA) technique for Dupuytren’s disease. The objective was to evaluate preliminary effectiveness and technical advantages with regard to contracture correction, complications, and recurrence rates.

## Materials and methods

Following approval from the Institutional Review Board, we reviewed patients who underwent our modified PNA technique. Patients with a palpable cord with moderate MCP joint contracture < 75° and PIP joint contracture < 15° and not beyond stage 2 Tubiana, were included in the study. Patients with severe PIP joint contracture greater than 15 degrees, severe MCP contracture greater than Tubiana stage 2, aggressive systemic disease, or not following through with postoperative physiotherapy were excluded from the study. Our cohort included 23 consecutive patients with 28 affected hands and 38 fingers (22 Ring, 6 Little, and 10 Middle), who underwent modified PNA to treat Dupuytren’s contracture (Table 1). The cohort consisted of 20 males and three females, whose mean age at presentation ranged from 47 to 78 years. Ten patients were agriculturists, four laborers, three businessmen, two fishermen, and one carpenter, and the remaining three females were homemakers. We classified the digits using the Tubiana classification system, which derives from Total passive extension deficit(TPED), based on the severity of contracture [4]. We determined the TPED by summing the values from each joint of the affected digit, resulting in an exclusive measurement for the involved digit. Thirty-three digits belonged to Tubiana stage 2 (45°–90°), and nine digits belonged to Tubiana stage 1 (0–45°). The preoperative status is documented through an assessment that includes the Heuston tabletop test [5] (Fig. 1C), a sensory assessment, and the documentation of total passive extension deficit (TPED) (Fig. 1D) prior to aponeurotomy. At presentation, the median metacarpophalangeal (MP) joint contracture was 40°(IQR 40°–60°), and the median proximal interphalangeal (PIP) joint contracture was 10°(IQR 0–10°). The average preoperative TPED was 50°(IQR 40°–60°). (Table 1) A senior author with level 4 expertise carried out all procedures, according to the Tang and Giddins criteria [6].

**Table 1:** Demographics of our patients with range of motion and postoperative URAM scores

Sl No	Age	Sex	Occupation	Side affected	Finger	Association	Preoperative ROM	Follow-up	ROM final	Final URAM
							MCP/PIP(TPED)		MCP/PIP(TPED)	
1	69	M	Agriculture	R	Middle, Ring	Smoking, Alcohol	30/0(30), 30/10(40)	18	0/0, 10/0(10)	0
2	55	M	Agriculture	L	Ring, Little	Diabetes, Alcohol	40/10(30) 30/0(30)	12	0/10(10), 10/0(10)	0
3	68	M	Agriculture	R	Middle	Alcohol	40/10(50)	13	10/0(10)	0
4	47	M	Fishing	R	Ring	Smoking	40/10(50)	16	0/0	0
5	59	M	Agriculture	R	Middle, Ring	Smoking, Alcohol	45/0(45), 30/10(40)	22	10/0(10), 0/0	0
6	62	M	Fishing	R	Middle	Nil	45/10(30)	28	10/0(10),	0
7	61	M	Agriculture	R	Ring	Diabetes	40/10(50)	22	0/0	0
8	48	M	Business	R	Ring, Little	Nil	50/10(60), 30/10(40)	17	10/10(20) 0/0	10
9	48	M	Carpenter	R	Ring	Smoking	55/10(65)	14	0/0	0
10	72	M	Laborer	R	Middle	Diabetes	45/20(65)	12	20/10(30)	15
				L	Ring		40/20(60)	11	10/0(10)	
11	69	M	Business	R	Ring, Little	HTN, Diabetes	50/10(60), 30/10(40)	14	10/0(10), 0/0	0
				L	Middle		40/20(60)	22	0/0	
12	65	F	Housewife	R	Ring	Hypothyroid, HTN	40/30(70)	26	10/0(10),	10
13	66	M	Agriculture	R	Ring, Little	HTN, Diabetes	45/20(65), 40/10(40)	21	10/0(10), 0/0	0
				L	Middle		45/10(55)	16	10/0(10)	
14	65	M	Laborer	R	Middle, Ring	Smoking, Plantar nodules	40/20(60), 45/10(55)	17	20/10(30) 10/0(10)	10
15	58	M	Agriculture	R	Ring, Little	Epilepsy	40/10(50), 50/0(50)	19	20/10(30) 10/0(10)	8
16	68	M	Agriculture	L	Ring, Middle	Smoking	40/0(40), 50/10(60)	14	0/0, 10/0(10)	0
17	57	M	Agriculture	R	Ring	Diabetes	40/0(40)	19	0/0	0
18	66	M	Agriculture	R	Ring	Hypertension	50/20(70)	18	10/0(10)	0
19	66	F	Housewife	L	Ring, Little	Diabetes	50/10(60), 30/0(30)	21	10/0(10), 0/0	8
20	59	M	Laborer	R	Ring	Nil	45/0(45)	28	0/0	10
				L	Middle		50/10(60)	24	10/0(10)	
21	73	M	Business	R	Ring	Nil	50/0(50)	26	10/0(10)	10
				L	Ring		40/0(40)	19	0/0	
22	63	M	Laborer	R	Ring	Diabetes	30/0(30)	14	0/0	0
23	52	F	Housewife	R	Ring	Epilepsy	45/20(65)	22	30/20(50)	16

### Surgical technique

**Fig. 2:**
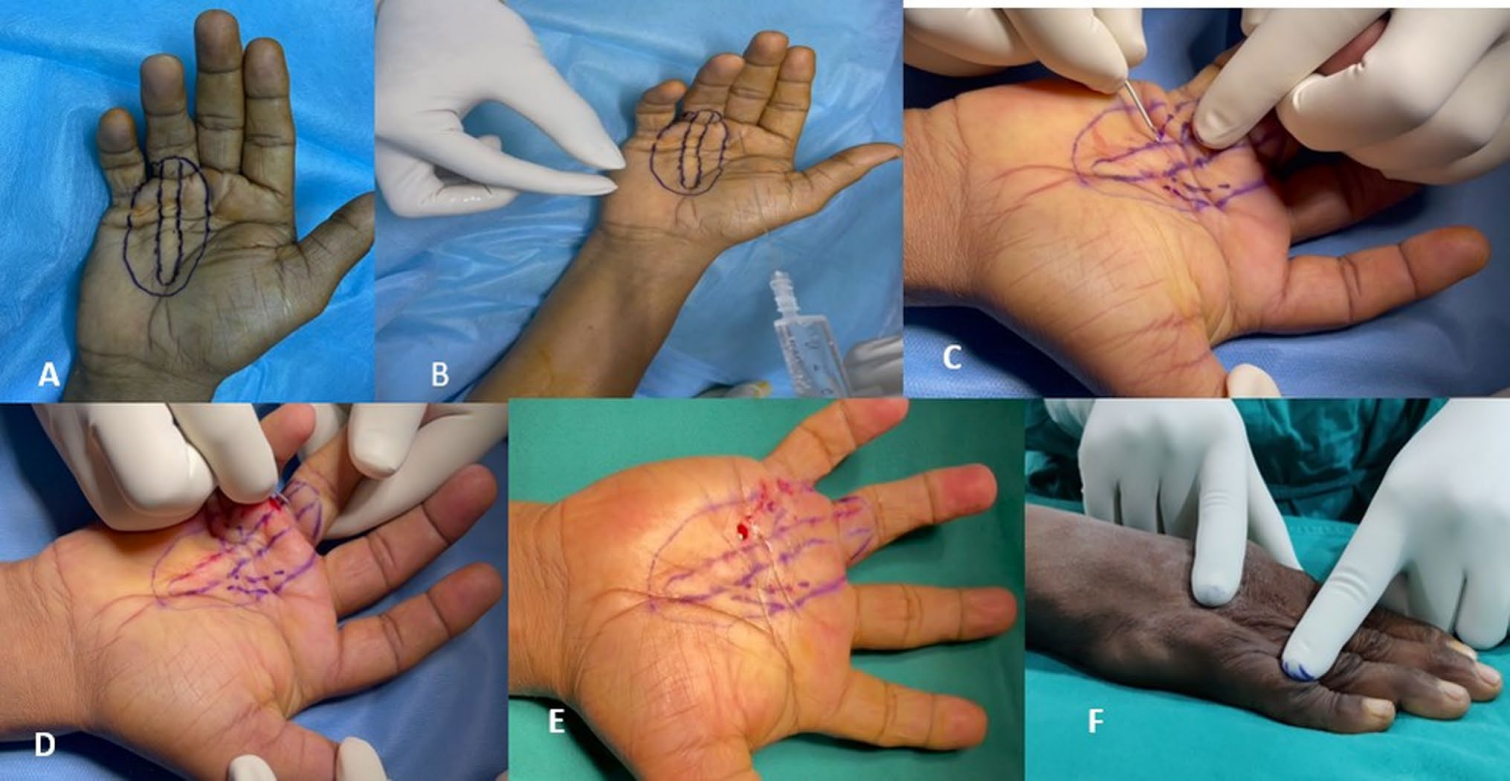
**A** Skin marking, **B** anesthesia provided with 10 mL syringe using a 29-gauge needle, **C** cord division in the palm using 18 gauge needle, **D** cord division near the web space and fingers, **E** complete release of the contracture, and **F** postoperative negative tabletop test

*Skin marking* (Fig. 2A): The initial step involves marking the palpable cord outlines up to the PIP joint.

*Anesthesia* (Fig. 2B): All procedures were conducted under wide awake local anesthesia with no tourniquet technique (WALANT) [7]. To induce surface anesthesia, the skin immediately above Dupuytren’s cord was injected with 5 ml of 2% lidocaine solution containing 0.1 ml of 1:1000 (1 mg/ml) epinephrine diluted in 5 ml of normal saline containing 1.2 ml of 7.5% sodium bicarbonate. This was performed using a 10 mL syringe and a 29-gauge needle. The injections are strategically administered intradermal or superficial subdermal to numb the skin without affecting the digital nerves. A minimal quantity of anesthetic (approximately 0.05 ml) was administered at each entry point. Avoidance of subcutaneous infiltration is recommended. Ensuring the precise dose of anesthesia and infiltration depth is crucial to prevent subsequent injury to the underlying digital nerves and arteries.

*Cord division in the palm* (Fig. 2C): Our technique seeks to achieve more significant cord division than previous methods of percutaneous aponeurotomy. Percutaneous fasciotomy using an 18-gauge needle is employed to precisely separate the affected cords in a controlled dorsovolar manner, which is in contrast to the previous technique. To prevent injury to the underlying neurovascular tissues, it is essential to maintain the midline and superficial position on the pretendinous cords [8]. Precise attention is given to the distal palm, specifically at the level of the flexor sheath, to prevent excessive penetration with the needle due to the relatively shallow position of the flexor tendons [8]. The portal is considered desolate if no opposition is encountered or if there is a supple or malleable feeling. After inserting the needle, it is essential to actively flex and extend the fingers to ensure that the needle has not penetrated the flexor tendon. We performed a dorsovolar cut with the bevel-up position of the 18 Gauge needle. The needle bevel walks on the cord from volar to dorsal initially and then cut is initiated from dorsal to volar. The release is initiated sequentially, starting from the proximal to the distal end, explicitly targeting the fibrous cords within the palm through various entry points. At all times, the surgeon keeps the bevel facing up. The cords are stretched by gently extending the injured finger during release. Taut cords should offer distinct grating audible feedback against needle-tip release [8]. Multiple entry points are employed, and the release process is carried out until no more areas of cord strain can be felt and all fibrotic skin is freed.

*Cord division near the web space and fingers* (Fig. 2D): Additional precautions must be taken when dealing with spiral and lateral cords that extend into the digits and the natatory cords in the web space. When the needle is inserted at these levels before dividing the cord, the patient is instructed to describe any abnormal sensations or paresthesia that might indicate that the needle is close to the digital nerve. If no symptoms are reported, the cord is meticulously separated delicately in a dorsovolar manner. The digits are then extended until the cords rupture entirely. (Fig. 2E).

*Postoperative tabletop test:* (Fig. 2F) The surgery is said to be complete when the patient can demonstrate a negative tabletop test on the table intraoperatively. This test confirms the successful release of the cords and ensures that the patient will have an improved range of motion postsurgery. A negative tabletop test is a crucial step in determining the success of the procedure and ensuring optimal outcomes for the patient.

### Rehabilitation and follow-up

**Fig. 3:**
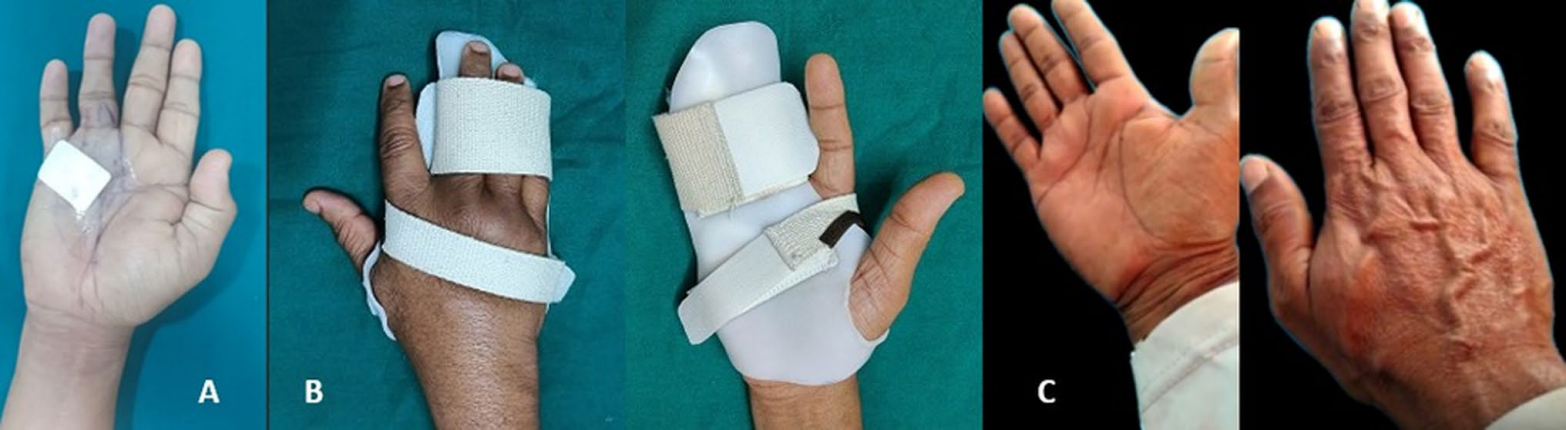
**A** Limited dressing to the operated area, **B** palm-based volar extension splint, and **C** no recurrence at 24-month follow-up

A volar extension slab for only the fingers is provided for one week, after which the patient was referred to a hand therapist. A palm-based finger extension splint is given to the patient, with the dressing limited to the operative area (Fig. 3A, 3B), which will be worn primarily at night for a minimum of 3 months. Patients with modest contractures and no skin problems typically do not require supervised therapy. Patients suffering from skin tears are instructed in wound care, including the application of antibiotic ointment and dressing to promote ongoing rehabilitation. We consider this a minor complication as part of the procedure, as they tend to heal by two weeks. The patient is instructed in active assisted and passive mobilization techniques, as well as passive stretching exercises, up to a comfortable level. Regular follow-up is recommended to monitor progress, make any necessary adjustments to the treatment plan, and keep a note of recurrences. Only patients with a minimum of 1-year follow-up were included in the study. At the final follow-up, the average metacarpophalangeal (MP) joint and the proximal interphalangeal (PIP) joint contractures were documented (Table 1) The average TPED with a mean percentage of correction of MCP and the PIP joint were also calculated. The Dupuytren’s contracture-specific URAM score was calculated at final follow-up. The URAM (Unité rhumatologique desaffections de la main) scale [9] is the most effective functional assessment tool for accurately evaluating the extent of disability caused by flexion contracture in Dupuytren’s disease patients. There are nine questions on the URAM scale that pertain to common daily activities; the questions’ relative ease of performance will determine their final score. Recurrence was calculated at final follow-up which was defined as the occurrence of contracture that exceeded 30° beyond the initial postperative total passive extension deficit (TPED) in an operated digit [10].

### Statistical analysis

The mean, median, and range of all the measured variables were calculated at the presentation and at the final follow-up. For skewed data, the median and inter quartile range (IQR) were calculated The preoperative and postoperative TPED were analyzed using a paired t test. Differences with *P* value < 0.05 were considered statistically significant.

## Results

The average duration of the follow-up period was 22 months, ranging from 12 to 28 months. At the final follow-up, the median metacarpophalangeal (MP) joint contracture was 10°(IQR 0 -10°), and the median proximal interphalangeal (PIP) joint contracture was 0°(IQR -0). (Table 1) The median TPED at the time of follow-up was 10°(IQR 0–10°), with a correction of 83%, which included 82% at the MP joint and 81% at the PIP joint. The difference between the preoperative TPED and postoperative values at the final follow-up was found to be significant calculated using paired t test (*P* < 0.05). The median URAM score at the final follow-up was zero with the inter quartile range of 0 to 10°.

We observed minimal complications, with five patients with skin wounds requiring daily dressing, which healed within 2 weeks, and 4 patients with transient neuropraxia. Four patients experienced recurrence, constituting up to 14.2% of our patients, but were able to manage their daily activities. The average TPED in the patients who experienced recurrence was 35 degrees. All patients returned to work at an average of 6-week postsurgery (range: 4–8 weeks). (Fig. 3C).

## Discussion

Percutaneous needle aponeurotomy (PNA) has gained popularity as a favorable choice due to its minimally invasive nature, making it a preferred initial treatment option for patients who are prone to recurrence [11]. In a compliant patient, needle aponeurotomy is a good option when there is a palpable cord causing finger contracture and the overlying skin is in good condition. Needle aponeurotomy is also suitable for elderly patients, especially those with associated morbidities [11]. However recurrence rates have been a problem and hence our modification. The critical variables that this study examined were the initial deformity, correction at the final follow-up, recurrence rate, complications, hand function, and disability.

Percutaneous needle aponeurotomy (PNA) can be performed as a day care surgery, as described by French rheumatologists Lermusiaux and Debeyre in 1979 [12]. As per their description, the involved finger was needled multiple times using a 25G needle attached to a 2 ml syringe while the patient was under local anesthesia provided by injecting 0.2 ml of 1% lidocaine [12]. Later, from 2007 to 2015, a group of Danish orthopedicians popularized this procedure, and it became known as the Silkeborg procedure globally [13]. This technique, which consequently weakens the cord, involves repeated needle-tip perforations into the cord, as well as careful pendulum cutting of the cord with a 25-gauge needle at a steady pace accompanied by passive stretching of the finger to rupture the Dupuytren cord [13].

Foucher and colleagues (2003) [14] conducted a study on 211 individuals who underwent percutaneous needle aponeurotomy using a 19-gauge needle whose bevel was used as a scalpel. The mean preoperative total passive extension deficit (TPED) was 65 degrees. At the interphalangeal level, the postoperative gain was 65%, and at the metacarpophalangeal joint level, it was 79%. The rate of reoperations was 24%. A subset of 100 patients, observed for an average period of 3.2 years, had a recurrence rate of 58% [14].

Fernando A. Herrera et al. (2015) [15] reviewed 512 digits from Dupuytren’s contracture patients who underwent treatment with a modified percutaneous needle aponeurotomy (NA) utilizing an 18-gauge needle in a sweeping fashion. However, the exact directions for beveling the needle and releasing the cord were not detailed. The mean preoperative TPED was 41 degrees. The mean TPED at the 4.5-month follow-up was 11 degrees, with a correction rate of 73%. Infection, delayed flexor tendon rupture, prolonged numbness, complex regional pain syndrome, and triggering were the noted complications. Recurrence was identified in 62 digits [15].

**Fig. 4:**
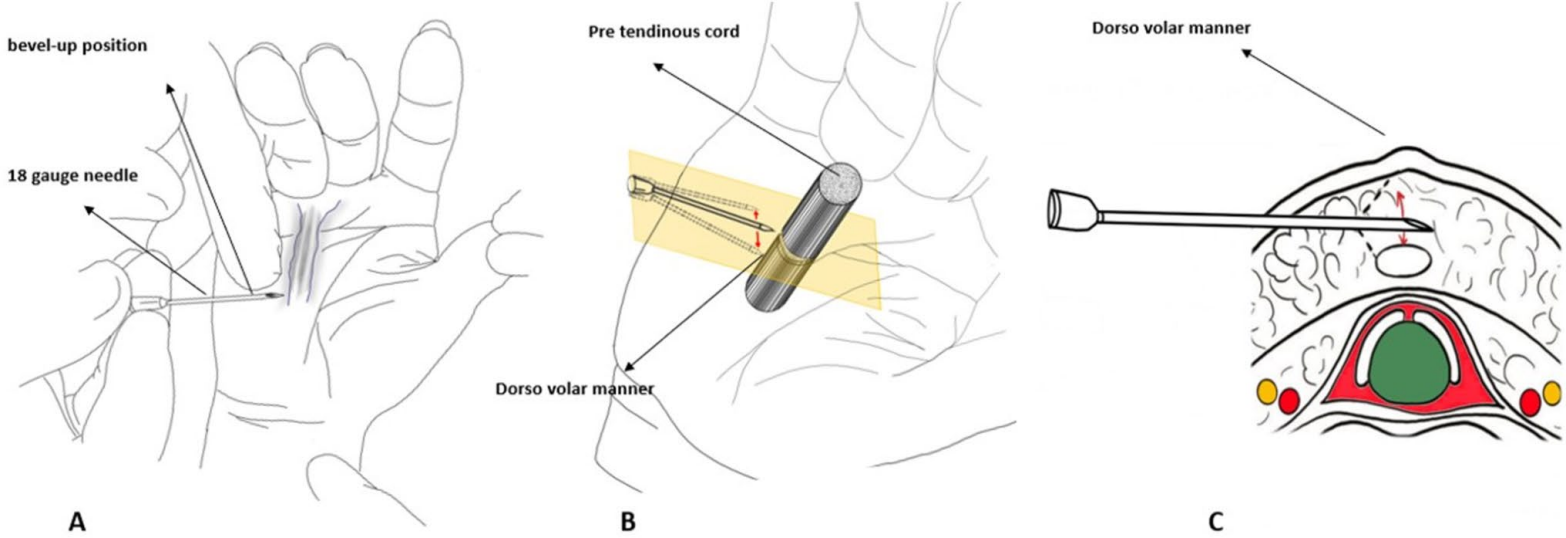
The diagram illustrates how to insert a 18 gauge needle parallel to the skin **A**, point the bevel upward **B**, and demonstrate both upward and downward (dorsovolar) **B** and **C** directions to cut the cords

Our technique differs from the previously used techniques, as we use a larger-bore 18-gauge needle under WALANT. The bevel is used to cut the cord facing upward in a dorsovolar manner, allowing for a more controlled and precise tissue-cutting process. (Fig. 4) This modification can result in more accurate and successful outcomes for patients undergoing Dupuytren cord release, particularly in the webspace and digits. Moreover, the larger-bore needle enables a more apparent appreciation of the tissue being cut, resulting in enhanced accuracy. The dorsovolar direction used decreases the risk of damage to the surrounding tissue and nerves, thereby improving the overall safety and precision of the procedure.

PNA recurrence rates ranging from 12 to 100% have been reported in published studies [11, 16]. Definitions and rates of contracture recurrence in patients undergoing invasive treatment of Dupuytren contracture were evaluated in the study by Paul M. N. Werker et al. (2012) [11]. Nineteen of the 21 studies that were examined had qualitative definitions of recurrence. Van Rijssen and colleagues [10]suggested a single quantitative criterion for recurrence being an increase in total passive extension deficit of more than 30° between immediate postoperative assessments and follow-up, which was also adopted in our study. We hypothesize that our approach, which achieves a more widespread disruption of the cord, may reduce the occurrence of recurrent contracture. However, longer follow-up is required to provide evidence in support of this hypothesis. In 2017, a systematic review by Krefter et al. [17] of 113 studies examined the incidence of complications associated with different treatment options for Dupuytren contracture. Following PNA, the review revealed a pooled complication rate of 19%. A significant finding in this study is the remarkably low occurrence of nerve injuries. Nevertheless, we have complete confidence in the reliability of our findings regarding the minor occurrence of nerve damage when we utilize the aforementioned PNA technique. Furthermore, the procedures were conducted by a single-skilled surgeon throughout the study duration, representing a certain competence level.

This study’s limitations must be considered when making decisions. Ours was a single-center study introducing a modification to an existing technique. This study was designed as a preliminary investigation to assess the potential benefits of our modified percutaneous needle aponeurotomy (PNA) technique. However, given the innovative and exploratory nature of the approach, our primary aim was to document early clinical outcomes before proceeding to larger scale studies rather than to establish definitive statistical conclusions. We included consecutive patients treated with the technique within a defined period as per the inclusion criteria, reducing selection bias but inherently limiting sample size. The follow-up period was short, averaging 22 months. An extended follow-up period would help detect delayed recurrence. While our findings demonstrate promising results, we acknowledge that further research with larger, controlled cohorts is necessary to confirm the effectiveness and long-term outcomes of this technique. In our study, the lower URAM scores observed postoperatively indicate significant short-term functional improvement. However, we acknowledge that the URAM questionnaire is primarily a functional assessment tool rather than a direct measure of patient satisfaction [9].

## Data Availability

No datasets were generated or analyzed during the current study.
